# Assessment of mouth breathing by Speech-Language Pathologists: an international Delphi consensus

**DOI:** 10.1590/2317-1782/20232022065

**Published:** 2023-05-29

**Authors:** Morgane Warnier, Leonor Piron, Dominique Morsomme, Christelle Maillart

**Affiliations:** 1 Department of Speech-Language Pathology, Research Unit for a Life-Course Perspective on Health and Education, University of Liège - Uliège - Liège, Belgium.

**Keywords:** Mouth Breathing, Child, Speech-Language Pathology, Delphi Technique, Clinical Assessment

## Abstract

**Purpose:**

mouth breathing (MB) has detrimental effects on children’s growth. Diagnosis of MB is possible through a multidisciplinary approach including Speech-Language Pathologist’s (SLP) assessment; however, SLPs currently have little to no defined selection criteria to determine the awake and habitual breathing pattern. This study aims at identifying relevant criteria for the assessment of the habitual and awake breathing pattern of preschool children, and developing a grid that would help SLPs diagnose MB in their clinical practice.

**Methods:**

A three-rounded online international Delphi process was conducted to achieve a consensus on the relevant items and their interpretation. Agreement was established through a Content Validity Ratio calculation. Based on the agreed items, we developed a grid through a scoring function.

**Results:**

Observing the child at rest (i.e., time spent with an open/closed mouth and position of the tongue/lips) was considered the most important criterion. The experts also considered that observing the breathing pattern while chewing (open/closed mouth) and after swallowing (i.e., air intake and open/ closed mouth just after swallowing) should provide relevant but secondary information in decision-making. We were able to establish a clinical grid based on those criteria.

**Conclusion:**

The Delphi procedure provided content-valid criteria and conditions of observation for the myofunctional SLP assessment of the awake and habitual breathing pattern in preschoolers. A clinical validation of the developed prototype grid should be conducted in preschool children to explore its effectiveness in the diagnosis of MB.

## INTRODUCTION

Mouth breathing (MB), formerly sometimes called oral breathing, is considered as a sign of orofacial myofunctional disorder and is gradually being recognized as an important health issue because of its comorbid conditions^(1)^. MB is particularly studied in children because it creates a self-perpetuating vicious circle between causes and consequences during growth. Indeed, the literature describes MB children as being at risk for swallowing and chewing disorders^(2)^, developing dental malocclusions^(3)^, impaired craniofacial growth^(4)^ and the onset of obstructive sleep disordered breathing^(5)^. Children who breathe through the mouth are also more likely to have attention deficits, working memory deficits, reading comprehension disorders and arithmetical difficulties, among others^(6)^. Speech sound disorders, in particular atypical placements for speech production like interdental lisp, are often seen in mouth breathers^(7)^. But most importantly, MB has been suspected to affect children’s quality of life^(8)^. The preschool period is a particularly good time for early diagnosis to prevent the onset of those comorbidities^(9)^.

Understanding the taxonomy of MB is a key step to facilitate diagnosis. It is well known that nasal breathing (NB) is the physiological pattern of breathing, yet MB is extremely rare^(10)^. For that reason, many authors consider mixed or oronasal breathing (OB) and MB as a whole^(8,10,11)^, whereas some authors distinguish the two conditions ^(7)^. It is currently not clear whether this distinction is useful for clinical purpose. When mouth breathing turns out to be the preferential and natural breathing pattern, the term habitual MB may be used regardless of the etiology of the habit (obstructive MB or functional MB)^(12,13)^. In addition, some authors refer to mouth breathing syndrome (MBS) when a set of signs and symptoms are completely or incompletely present, e.g., craniofacial features^(11,14)^. Breathing through the mouth is especially problematic when it becomes chronic, manifesting itself over the long term. A period of 6 months or more is often considered as a benchmark^(5)^. MB can occur either during sleep and/or while awake. Current literature doesn’t always make the difference and both conditions are generally combined^(1,11,13)^. Therefore, very little is known about how they interact. Sleep breathing pattern is more studied, mainly for its presence in the continuum of sleep-disordered breathing^(5)^ whereas less information is available on awake mouth breathing.

A multidisciplinary approach is commonly indicated to confirm the diagnosis and identify the characteristics in a syndromic perspective. The team commonly includes an orthodontist, an otorhinolaryngologist (ENT), a physiotherapist and a Speech-Language Pathologist’s (SLP)^(1,2,15)^. The orthodontist relies mainly on morphological and dental characteristics^(4)^; the ENT is able to differentiate obstructive MB (e.g., because of allergic rhinitis) from functional causes (e.g., by persistence after adenotonsillectomy) thanks to endoscopic examinations^(16)^ and the physiotherapist identifies head and body posture characteristics^(17)^.

The SLP takes part in the diagnosis with a full myofunctional examination and provides information about the awake and habitual breathing pattern from a functional perspective^(2)^. Among the published myofunctional protocols that include the assessment of breathing patterns are the Orofacial Myofunctional Evaluation with Scores (OMES)^(18)^, the Expanded Orofacial Myofunctional Evaluation with Scores- (OMES-E)^(19)^, which is a more complete version of the OMES, and the MBGR protocol^(20)^. Whether in these protocols or in the clinical practice, there are currently no precise criteria to guide the clinician in the decision-making process regarding the awake and habitual breathing pattern. The choice generally relies on the clinical expertise in the field and the experience with previous MB patients. Objective diagnostic methods exist, such as the CO_2_ sensor used by Fujimoto et al.^(12)^, but to our knowledge none has been conducted in children.

In addition, the contexts to observe the habitual breathing pattern have been little explored. Some authors have suggested that the breathing pattern could be assessed at rest, for example during a continuous five-minute condition^(12)^. De Felício also suggests that awake and habitual breathing could also be observed during chewing^(19)^. Knösel and colleagues hypothesized that it could be observed after swallowing^(21)^. In the clinical practice, the contexts in which to observe the child in order to determine his/her awake and habitual breathing pattern are often left to the discretion of the SLP.

In sum, the multidisciplinary diagnosis is mainly based on a set of features that define the MBS rather than the habit of MB itself^(15,22)^. It is currently difficult to determine the categories of classification of the breathing pattern, but also to define the contexts of observation and the criteria used by SLPs to determine the child's awake and habitual breathing pattern. Moreover, it seems necessary to define the criteria that SLP use to guide their choice in determining the awake and habitual breathing pattern. These criteria are particularly important for preschool children since the early onset of MB may show more detrimental effects on growth^(5)^. This would provide clear and reproducible recommendations to determine the habitual breathing pattern in young children.

## METHODS

The present research was approved by the Research Ethics Committee of University of Liège, Belgium (protocol n°1819-35). Participants gave their full consent to enter the study.

### Objectives

The first objective of this study was to achieve a consensus on the relevant items and their interpretation to classify the breathing pattern in preschool children. Specifically, we wanted to identify:

- The contexts in which to observe the children’s breathing so that they are representative of the habitual breathing behavior (level A); as well as (b) the conditions of the child’s observation
  - Within each context, the general relevant criteria to observe (level B)
  - Within each criterion, the tangible manifestations or signs that would help to classify the breathing pattern (level C)

**Figure 1:**
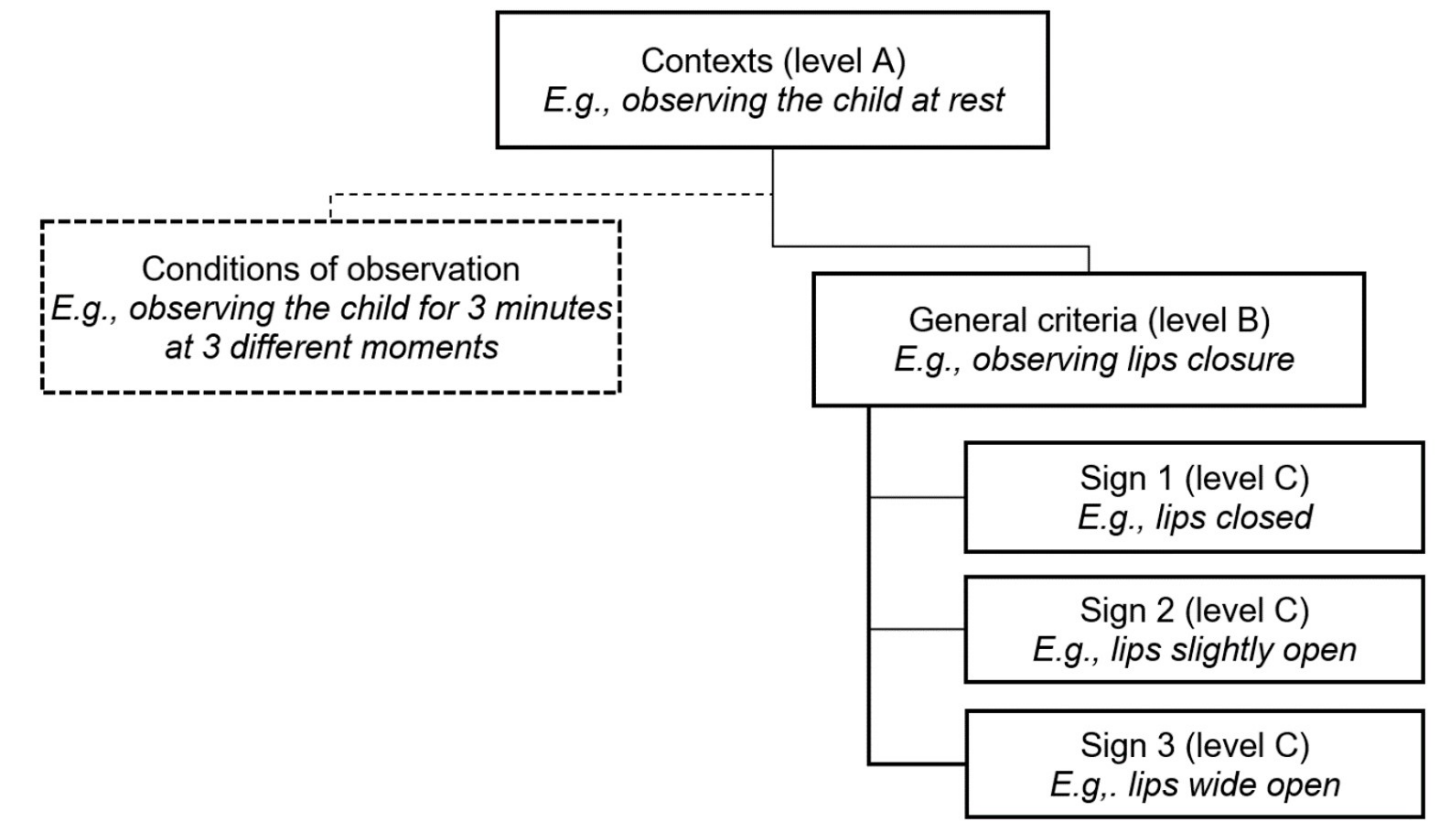
Organization of items aiming to determine the breathing pattern in preschool children

Figure 1 schematically represents these different objectives and their organization. An example is assigned to facilitate understanding.

After establishing this consensus, the second main objective was to develop a content-validated clinical grid that could be used as part of the SLP’s clinical assessment to classify the awake and habitual breathing pattern of preschoolers.

### Study design

SLPs completed an iterative online survey according to a Delphi technique, which is a facilitating process to anonymously reach group consensus through multiple rounds^(23)^. Three rounds took place in the present study. Data were collected through multiple choices questionnaires and participants were left with the possibility of leaving comments under each item. This survey was held online on a protected platform developed at the University of Liège, Belgium. The participants received an email invitation to take part in each round and were free not to participate or to stop the study at any time. Participants were not required to take part in all rounds. Data were collected anonymously.

### Participants

Both academics and clinicians were invited to participate in the study as a heterogeneous panel is recommended for a Delphi process^(24)^. Participants were selected either for their relevant publications about MB, for their lecture about MB at international congresses or for being internship supervisors, instructors or teachers in the field of orofacial myology/myofunctional sciences. Only SLPs were included since this study focuses on the SLPs’ assessment in the diagnostic process. The moderators made sure to include an international panel of experts, as advised for a Delphi process^(24)^. This was possible because the classification of the breathing pattern is not subject to linguistic constraints. Represented countries were Australia, Austria, Belgium, Brazil, Canada, Chile, France, Italy, United Kingdom and United States of America. Participants were recruited by email, 32 experts were approached and 18 agreed to participate. The avarage professional experience was 19.28 years, ranging from 4 to 40 years. The majority of participants were clinicians, but three participants also had a research activity. Out of the 18 experts who agreed to participate, 14 experts actually participated in the first round, 15 experts participated in the second round and 9 in the last round. Responsive rate was respectively 44%, 46.8% and 28%.

### Procedure

#### Baseline items

Before the first round, a list of core items was established to create the baseline protocol proposal. This list was based on a comprehensive review of the current literature found on Medline, Scopus, as well as through a hand search, Google scholar search and references of included articles. The search strategy including the MeSH and text words applied in the initial search was: ((habitual OR functional) AND ((mouth OR oral OR open) AND breathing) AND (sign OR symptoms OR diagnosis OR screening). No age restrictions were applied to the review process due to the small number of specific studies on preschool children. Baseline items were selected from articles or test protocols when meeting three main inclusion criteria, i.e.:

- considered as functional criteria that could help with the detection of the awake and habitual breathing pattern,
- considered as suitable for the SLP’s myofunctional assessment or when used in myofunctional assessment^(18,19)^, and
- considered as empirically relevant^(2,12,15,25)^ or employed as criteria for selecting a population of mouth breathers^(11,18)^.

As we focused on the functional assessment of habitual breathing pattern rather than the diagnosis of MBS, items were excluded if they based the breathing pattern classification on:

- anatomical or physical factors, e.g., long face or dark circles
- supposed consequences of MB, e.g., malocclusion
- causes of MB, e.g., nasal obstruction

The first author, who has clinical and research experience in the field, also clarified the items when they were insufficiently precise. Baseline items were written in simple and accessible English, without ambiguity and in a neutral manner^(26)^.

#### Round 1

The first round of the Delphi process aimed at identifying and validating relevant criteria for the assessment of the awake and habitual breathing pattern in preschoolers. The survey consisted of three questionnaires. The first questionnaire included general information about the participants such as their years of experience, country or language. In the second questionnaire, experts were asked to share assessment criteria from their personal experience. Participants were then invited to identify the main categories to classify the breathing pattern (e.g., NB and MB or NB, MB and OB). In the last questionnaire, participants were asked to judge each baseline item from each level (A, B and C) as *essential*, *nonessential* or *essential but imperfect/incomplete*. Participants could suggest the removal or revision of each item in the comments, provide more details or add new items in comments. Agreement on items was calculated through a Content Validity Ratio (CVR) according to this Equation 1 ^(27)^: 

$\frac{n_{e}-\frac{N}{2}}{\frac{N}{2}}$(1)

(*n_e_* is the number of experts who judge item as *essential* and *N* is the number of experts who participated in the round). 

CVR was then compared to the Wilson adapted reference table for a unilateral test (α=0.05). If the item did not reach sufficient CVR, it was reworded following the experts’ comments and the adapted item was proposed in the next round.

#### Round 2

The second round pursued the exact same goals as the first one but with the aim of defining the final items. The exact same methodology was applied to the adapted list of items, including modified and added items following the controlled feedback of the first round. Agreement on items was calculated in the same manner. This time, when an item happened to be under the CVR threshold, it was definitively removed from the list. The removal of an item automatically resulted in the removal of items from underlying levels. Overall content validity of definitive items validated through the CVR agreement was measured through the S-CVI/Ave^2^ method. In the particular case where CVR was not sufficient but the majority of the experts indicated in their comments that the item was essential, we used the qualitative validation method from Boateng et al.^(28)^ to reword the final item.

#### Round 3

The third and last round aimed at obtaining sufficient information to organize validated items in an assessment tool. This third round was also conditioned by previous answers. It was divided in three parts to establish a link between each level.

**Figure 2:**
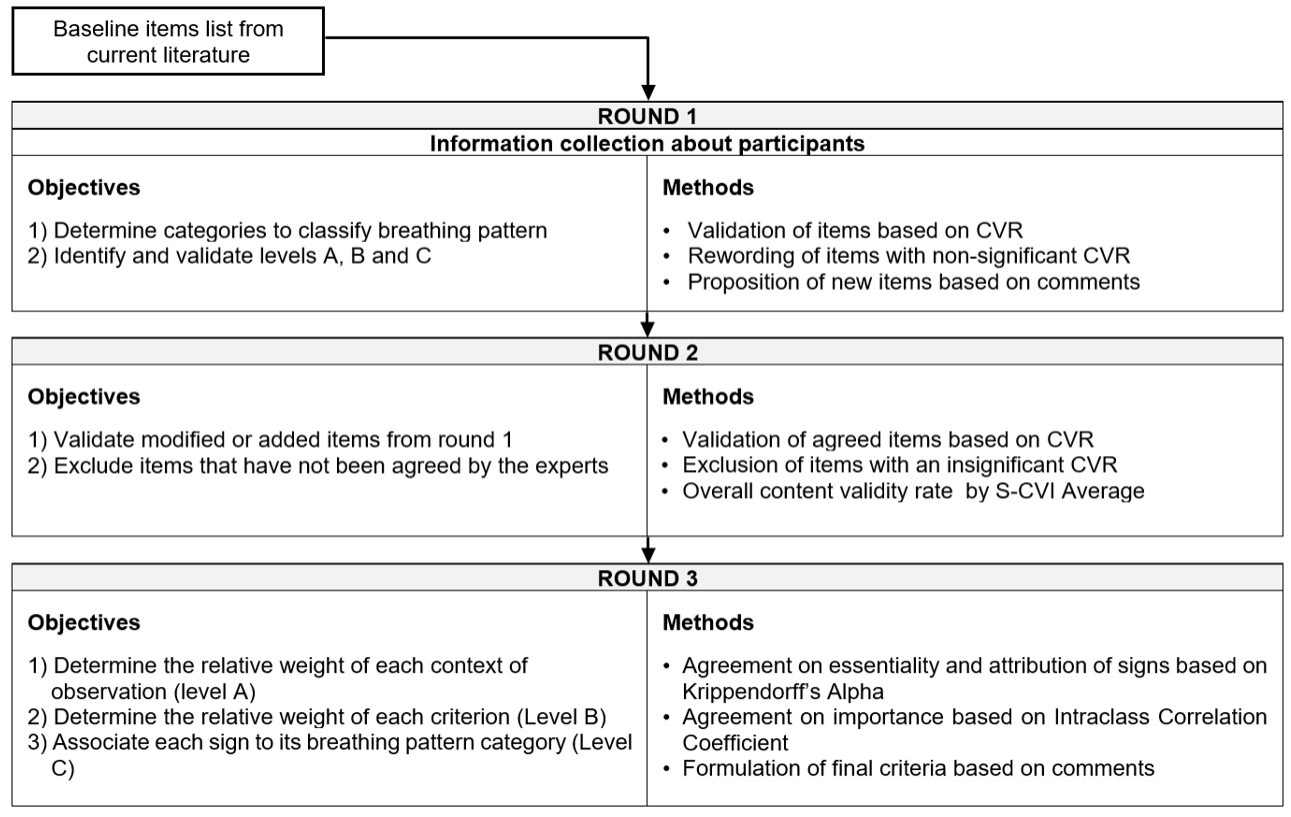
Objectives and methods of analyses for each round within the Delphi procedure

1. We asked participants to judge contexts (level A) as *fundamental* (highly necessary to define the breathing pattern) or *secondary* (information brought are less decisive to define the breathing pattern). Agreement between experts was calculated through a Krippendorff’s Alpha for nonparametric data.
2. We asked participants to judge relative importance of each criterion (level B) via a gauge from 0 to 10. Subsequent signs received the same ranking. Agreement between experts was calculated through a two-way mixed effects Intraclass Correlation Coefficient (ICC) for multiple raters^(29)^. Relative importance of criteria was calculated based on the mean and standard deviation.
3. We finally asked participants to match each sign (level C) to one or more breathing pattern. If one sign was associated with two patterns, we considered the most significant association (*p* < .01) to be the main profile and less significant association (*p* < .05) to be the second profile. Agreement between experts was calculated through a Krippendorff’s Alpha for nonparametric data. To assess the association between each sign and their matched pattern, a Wilcoxon signed-rank test was used. This test provides a probability that the results are due to chance or not.
  Figure 2 displays the three rounds of the Delphi procedure and their respective objectives in this study and summarizes the methodology that was used.

### Clinical grid

The clinical grid includes all items that were validated during the Delphi process. An automatic calculation is used to determine both a main and a secondary breathing pattern. The outcome relies on weight coefficients attributed to each sign based on their importance and the breathing pattern(s) previously assigned by the experts. The calculation for the weight coefficient for each criterion (level B) was based on central tendency measures. This option was chosen as we expected agreements between experts^(29)^. A scoring function was computed to calculate weight coefficients. The chosen method transformed each criterion’s central tendency scores according to the following function (Equation 2): 


$$coefficient\;=\frac{mean^{2}}{Standard\;Deviation+4}$$ (2)

The mean was squared, so that it became a quadratic function, which increases the variations of the score for an equal variation of the mean. As we expected an agreement between experts, we also expected that some standard deviation values may fall below 1 and reverse the variation function. So, we decided to anticipate this potential risk by enhancing the value of the standard deviation. The number 4 appeared to be a good choice for managing this risk while highlighting the respective impact of each criterion in the decision-making process.

## RESULTS

### Baseline items

The literature search allowed us to select 31 articles to establish the baseline items. Baseline items and associated references are presented in Appendix 1.

#### Round 1

The first questionnaire reached a consensus on the classifications of the breathing pattern. Experts agreed to classify the breathing pattern into three categories: NB, MB and OB. CVR reached .57 and was above the critical value of .44 (N = 14). Then, out of the overall 49 items proposed, 28 were content validated. Out of the four contexts of observation (level A), three were validated: observation at rest, after swallowing and during mastication. Four criteria (level B) and 20 signs (level C) were validated. None of the observation conditions were validated at this time. Experts also suggested in their comments to add one criterion (level B) and subsequent signs: the position of the tongue while the child breathes at rest. All the non-validated items were analyzed and modified according to the experts’ comments. The added and the modified items were proposed in the second questionnaire for a new attempt at consensus.

#### Round 2

Out of the 29 proposed (23 modified and 6 added), 16 items reached consensus with a CVR value above the critical value of 0.425 (N = 15). Items were removed from the list if they did not reach the CVR threshold and were considered as non-essential in the comments. The first removed item (level A and subsequent items) was “*Encouraging or forcing the child to breathe through the nose (forced nasal breathing) is relevant to determine the awake and habitual breathing pattern*” (CVR = .14; below critical value of .425). Experts justified their decision on the basis that noises are a symptom of obstruction rather than a reflection of habitual MB. The second item (level B and subsequent items) rejected by experts was “*Observing more than one occurrence of some habits/behaviors (finger sucking, nose itching, lips playing) during the entire observation is relevant to determine the awake and habitual breathing pattern*” (CVR = .33; below critical value of .425). Again, the experts considered that these signs were more predictive behaviors or causal factors of MB. Last rejected item was (level B and subsequent items) “*Knowing that the child does not present any medical condition, hearing noisy breathing at rest is relevant to determine the awake and habitual breathing pattern”* (CVR = -.20; below critical value of .425).

The conditions of observation linked to the contexts of breathing, swallowing and chewing did not reach the CVR threshold either. The experts considered them as essential in the comments but did not agree on the wording of the conditions. For this reason, they were retained and rewritten following the experts’ suggestions. Baseline conditions, experts' comments and reworded conditions are presented in Appendix 2.

Finally, we rejected two signs (level C) linked to the chewing context : *"Observing the child chewing with his/her mouth open” ; “Observing the child chewing with his/her mouth closed*" because they were too similar to the signs of the criterion "*The time spent chewing with an open or a closed mouth "*.

Description of all items from the first and the second round with their respective CVR are detailed in Appendix 3.

#### Round 3

##### Importance and weight of items

Experts judged the context (level A) of “breathing at rest” as *fundamental* and the contexts of “breathing after swallowing” and “breathing while chewing” as *secondary* (α = .21, fair agreement). Order of importance of the criteria (level B) are shown in Table 1. Agreement for the order of importance was good (ICC = .8).

**Table 1:** Relative importance of each criterion (level B) from 0 to 10 and their global mean and standard deviation

	Time spent with open/closed mouth	Tongue posture	Lips position	Tongue position after swallowing	Time spent chewing with open/closed mouth	Air intake after swallowing
Expert1	10	10	10	5	3	3
Expert2	7	7	10	8	5	8
Expert3	5	5	5	5	5	5
Expert4	10	9	9	8	10	8
Expert5	10	10	5	6	7	7
Expert6	10	10	5	5	5	5
Expert7	10	7	10	6	6	6
Expert8	8	8	8	8	6	8
Expert9	9	9	9	7	7	3
**Mean (SD)**	**8.78 (1.79)**	**8.33 (1.73)**	**7.89 (2.26)**	**6.44 (1.33)**	**6.00 (1.94)**	**5.89 (2.03)**

##### Association of signs with the breathing pattern

Agreement on the association of signs with the breathing pattern was fair (α = .22). After the Wilcoxon for signed rank test (for a mean or median equals to 0), each sign was linked to one or two breathing pattern(s). Main and second profiles and their respective association are detailed in Table 2.

**Table 2:** Signs (level C) and matched breathing pattern(s)

	NB	OB	MB
SIGNS	n (p)	n (p)	n (p)
Observing fully closed lips for more than half of the time	8 (0.0078)**	4 (0.1250)	0 (1)
Observing slightly open lips for more than half of the time	2 (0.5)	8 (0.0078)**	3 (0.25)
Observing half-open lips for more than half of the time	0 (1)	8 (0.0078)**	6 (0.0313)*
Observing wide open lips for more than half of the time	0 (1)	4 (0.1250)	8 (0.0078)**
Not observing a main pattern (sometimes the lips are open, sometimes the lips are closed)	3 (0.25)	9 (0.0039)**	2 (0.5)
Observing an upper tongue position for more than half of the time	9 (0.0039)**	3 (0.25)	0 (1)
Observing a low tongue position for more than half of the time	3 (0.25)	5 (0.0625)	6 (0.0313)*
Observing a low and forward tongue position for more than half of the time	0 (1)	3 (0.25)	9 (0.0039)**
Not observing the tongue position (because of closed lips) for more than half of the time	8 (0.0078)**	4 (0.1250)	1(1)
Observing an open mouth posture for more than half of the time	2 (0.5)	6 (0.0313)*	8 (0.0078)**
Observing the mouth closed for more than half of the time	8 (0.0078)**	6 (0.0313)*	0 (1)
Observing an open mouth posture for the entire observation time	0 (1)	2 (0.5)	9 (0.0039)**
Observing the mouth closed for the entire observation time	9 (0.0039)**	2 (0.5)	0 (1)
Observing an open mouth posture for more than half of the chewing occurrence	3 (0.25)	6 (0.0313)*	5 (0.0625)
Observing the mouth closed for more than half of the chewing occurrences	8 (0.0078)**	7 (0.0156)*	0 (1)
Observing an open mouth posture for all the chewing occurrences	3 (0.25)	4 (0.1250)	8 (0.0078)**
Observing the mouth closed for all the chewing occurrences	9 (0.0039)**	3 (0.25)	0 (1)
Observing the child breathing through his/her mouth just after swallowing	0 (1)	7 (0.0156)*	6(0.0313)*
Observing the child breathing through his/her nose just after swallowing	8 (0.0078)**	3 (0.25)	0 (1)

* p < .05;

** p < .01

**Caption:** NB = nasal breather; OB = oronasal breather; MB = mouth breather.

### Final consensus

At the end of the Delphi process, we were able to extract three main observation contexts (level A) as well as four conditions of observation, six criteria (level B) and twenty-one signs (level C). The first context (level A), judged by far as the most important by experts, consists in observing the child while at rest in a spontaneous and stress-free context. For example, while the child is watching a movie, drawing or playing without talking. We initially proposed to observe the child for five consecutive minutes^(12)^, but experts mentioned in their comments that it was more interesting to observe the child in several contexts and at different non-consecutive times. We therefore rewrote the final condition taking into account their suggestions: “*To observe the child for three consecutive minutes at rest in three different situations*”. Within this first context, the proportion of time spent with lips open (level B) was readily accepted by the experts and was considered as the most important criterion. Experts also suggested adding a criterion on tongue position (level B). For instance, observing a low and forward position of the tongue for more than half of the observation time was associated with MB. The position of the lips was also considered as a relevant criterion (e.g., wide-open lips were associated with MB whereas slightly open lips were associated with OB and half-open lips were first associated with OB and then to MB). However, the position of the tongue has been found to be more important.

Two other contexts of observation (level A) were accepted: the observation of the breathing pattern while chewing and the observation of the air intake just after swallowing. These contexts and their respective items were considered to have a much lower weight and importance than observing breathing at rest. During chewing, the experts considered that at least three bites of a biscuit were sufficient to observe the breathing pattern. Experts suggested that the posture of the mouth was the only factor to observe. For air intake after swallowing, the experts considered three swallowing movements of a solid or liquid to be sufficient. They considered closed lips and nose breathing just after swallowing to be consistent with the NB profile.

All retained final items, their conditions of observation, their order of importance and the main and secondary pattern attributed are summarized in Table 3.

**Table 3:** Summary of the main results

Items	Order of importance	Main pattern	Second pattern
Observing the child breathing at rest			
*Observe the child for 3 consecutive minutes at rest in 3 different resting situations (e.g., watching a movie, drawing, playing quietly or threading beads) and at different moments of the assessment’s situation.*			
The time spent breathing at rest with a closed or open mouth			
**- Observing an open mouth posture for more than half of the time**	**1**	**MB**	**OB**
**- Observing the mouth closed for more than half of the time**		**NB**	**OB**
**- Observing an open mouth posture for the entire observation time**		**MB**	
**- Observing the mouth closed for the entire observation time**		**NB**	
At rest, the position that the tongue occupies for more than half of the time			
**- Observing an upper tongue position for more than half of the time**	**2**	**NB**	
**- Observing a low tongue position for more than half of the time**			**MB**
**- Observing a low and forward tongue position for more than half of the time**		**MB**	
**- Not observing the tongue position (because of closed lips) for more than half of the time**		**NB**	
At rest, watching how open the lips are for more than half of the time			
**- Observing fully closed lips for more than half of the time**	**3**	**NB**	
**- Observing slightly open lips for more than half of the time**		**OB**	
**- Observing half-open lips for more than half of the time**		**OB**	**MB**
**- Observing wide open lips for more than half of the time**		**MB**	
**- Not observing a main pattern (sometimes the lips are open, sometimes the lips are closed)**		**OB**	
Observing the child's breathing while chewing			
*Observe the child eating 1 or 2 biscuits (at least 3 bites)*			
The time spent chewing with an open or a closed mouth			
**- Observing an open mouth posture for more than half of the chewing occurrences**	**5**	**OB**	**MB**
**- Observing the mouth closed for more than half of the chewing occurrences**		**NB**	**OB**
**- Observing an open mouth posture for all the chewing occurrences**		**MB**	
**- Observing the mouth closed for all the chewing occurrences**		**NB**	
Observing the child's air intake after swallowing			
***Observe the child drinking a small glass of water (at least 3 sips) and watch the air intake after each swallow*** *Observe the child eating 1 or 2 biscuits (at least 3 swallows) and watch the air intake after each swallow*			
The rest position of the mouth just after swallowing (observing that after swallowing, the child directly opens the mouth or keeps it closed)			
**- Observing the mouth closed just after swallowing in most cases**	**4**	**NB**	
**- Observing a mouth opening just after swallowing in most cases**		**MB**	**OB**
The air intake pattern just after swallowing (through the mouth or through the nose)			
**- Observing the child breathing through his/her mouth just after swallowing**	**6**	**OB**	**MB**
**- Observing the child breathing through his/her nose just after swallowing**		**NB**	

NB = nasal breathing, MB = mouth breathing, OB = oronasal breathing

### Clinical grid

The grid offers a decision on a main as well as a secondary awake and habitual breathing pattern: NB, MB and/or OB. Weight coefficient takes into account the importance given to the signs, displayed in Table 1, and the breathing pattern(s) assigned to them, displayed in Table 2. The weight coefficients obtained through the scoring function are shown in Table 4.

**Table 4:** Weight of the relative importance of each item (level B) for the clinical grid creation

**Criterion**	**Weight**
**The time spent breathing at rest with a closed or open mouth**	13,31
**At rest, the position that the tongue occupies for more than half of the time**	12,11
**At rest, watching how open the lips are for more than half of the time**	9,94
**The rest position of the mouth just after swallowing (observing that after swallowing, the child directly opens the mouth or keeps it close)**	7,78
**The time spent chewing with an open or a closed mouth**	6,06
**The air intake pattern just after swallowing (through the mouth or through the nose)**	5,75

**Figure 3:**
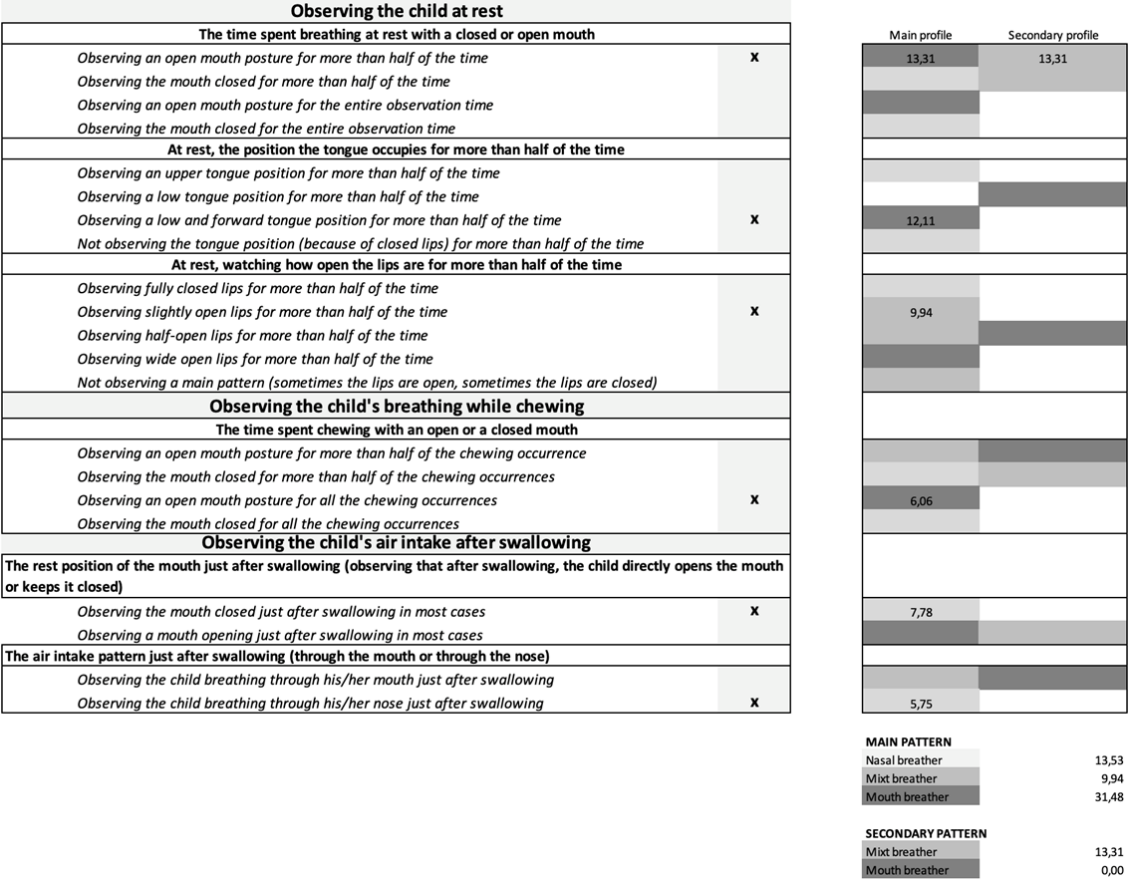
Prototype of the clinical grid completed with a fictional profile

Only one sign can be selected within each criterion to describe the child’s behavior. The clinician will select a total of six signs as they are mutually exclusive. When selecting a sign, its respective weight coefficient and assigned breathing pattern(s) influence the final score. Main and secondary patterns will ultimately come out according to the weight coefficient and the breathing pattern linked to the six signs selected. The prototype of this clinical grid, completed with a fictional profile, is displayed in Figure 3.

## DISCUSSION

The importance of early management of MB led us to restrict the age range to the preschool period^(13)^. We also decided to focus on the diagnosis of the awake and habitual breathing pattern by referring to the way the child breathes naturally and without constraint in everyday life. Indeed, habitual breathing is the reflection of the natural orofacial function, which is at the center of the SLP’s scope of practice^(2,13,15)^.

Given the lack of clear recommendations and valid clinical tools for SLPs, this study aimed to achieve an international consensus on the relevant items and their interpretation used to distinguish and classify the awake and habitual breathing pattern in preschool children. Our main results showed that observing the child at rest (i.e., time spend with an open/closed mouth and position of the tongue/lips) was considered the most important criterion. The experts also considered that observing the breathing pattern while chewing (open/closed mouth) and after swallowing (i.e., air intake and open/ closed mouth just after swallowing) should provide relevant but secondary information in decision-making. On the basis of this consensus, we have developed a clinical grid to guide clinicians in their decision-making.

### Determine the habitual awake breathing pattern in preschool children

Many authors had previously considered MB and OB as part of the same classification^(1,11,18)^. However, experts who participated in this study agreed to differentiate between NB, MB and OB, as it previously seen in the literature^(12,20,30)^. This suggests that the breathing pattern should be best described as a general and predominant trend^(10,30)^, which fluctuates over time. Some children therefore tend to fall in between the two main patterns.

Observing the child while at rest is very common to assess the breathing pattern^(12,19)^. However, experts of this study brought new information by suggesting the breathing function should be observed in several different contexts and at non-consecutive times to take into account fluctuations over time. These recommendations should help to corroborate the observed information and better represent the preferential and natural breathing pattern.

The most frequently observed criterion in the literature is the position of the mouth and/or lips^(2,13,15,19,30)^. The position of the mouth and lips was considered as relevant but again the proportion of time spent with lips/mouth open was considered as a major factor. Very few studies had then included this notion of time^(12)^, although it seems essential as the breathing pattern should be considered as a general trend. Experts also suggested adding a criterion on tongue position, as often included in SLP’s assessment^(15)^. This is consistent with the idea that the base of the tongue plays a role of sealing while NB^(12)^ and undergoes an adaptive response to free oropharyngeal space while MB^(2,30)^. As tongue position was considered more important than the lips position, a child with open lips (irrespective of amplitude) but a tongue in a high position would match with a NB profile. Experts also associated the sign “slightly open lips” with OB, whereas “half-open lips” was primarily associated with OB and secondarily with MB. Milanesi et al.^(16)^ previously underlined the need to consider the range of lips opening in the identification of MB. Our results allowed us to go further by specifying the breathing patterns associated with these signs.

The observation of the breathing pattern while chewing and the observation of the air intake just after swallowing as well as their respective items were considered relevant, as previously suggested by some authors^(18,21)^. However, both contexts were considered to be of secondary significance. It is important to note that this item does not correspond to assess the quality of chewing or swallowing function. Indeed, although MB may impact these functions^(2)^, assessing the quality of chewing and swallowing functions does not directly inform the child's preferred and habitual breathing pattern.

Our study supports prior findings to assess the breathing pattern during functional assessment. However, we also found important differences with previous studies. First, within the context of observation at rest, experts judged the observation of noisy breathing as irrelevant. These results are in disagreement with the study of Valera et al.^(30)^, who observed inspiratory noises in 60% of preschool mouth breathers. Experts of this study justified their decision on the basis that noises are a symptom of obstruction rather than a reflection of habitual MB. Second, all signs linked to habits/behaviors like finger sucking, nose itching or lips playing were considered irrelevant, as these signs were more predictive behaviors or causal factors of MB. Third, based on the literature review, we had initially proposed to observe the child during induced or forced NB^(19,22,25)^. This criterion was considered non-reliable to describe the habitual mouth breathing. Assessing the child’s ability to breathe through the nose has been shown to effectively differentiate obstructive MB from functional MB. However, many children who usually breathe through the mouth are quite capable of breathing through their nose when forced to do so^(25)^. Same limitations could apply to tests used by SLP in the myofunctional assessment, for instance lip seal or water retention tests^(22)^.

### Limitations and strengths

This study has some limitations. First, the number of experts who participated in the Delphi process barely reached the minimum threshold generally recommended for the first two rounds (at least 10 participants)^(23)^. The third round reached only nine participants. It is also important to note that the grid is currently at a prototypical stage and cannot be used as it stands in practice yet. Though it has been content validated, it now requires clinical validation in terms of internal consistency, inter-rater reliability, sensitivity and specificity in comparison to an objective tool, such as the one used by Fujimoto and colleagues^(12)^ As mentioned earlier, the notion of chronicity is central to the diagnosis of MB^(5)^. It will therefore be essential to consider it if the grid proves to be valid. Finally, the presence of open lips position is not systemically considered to be sufficient to prove the presence of MB^(12,13)^. A child who breathes through the mouth has automatically the lips opened; on the contrary, a child with open lips could as well breathe through the nose. Despite the fact that this grid provides more information, such as the position of the tongue, clinical visual examination alone may not be reliable and representative enough to establish diagnosis. Hence, a multidisciplinary approach remains essential.

On the other hand, the strong methodology provided by the Delphi process adds reliability to the development of clinical guidelines in the field of SLP specialized in orofacial myology/myofunctional sciences. Each item is based on clear recommendations and well-defined observation contexts taking into account the constraints peculiar to the SLP's assessment, such as limited session time. If validated, this grid would facilitate early diagnosis of awake and habitual MB and initiate early management to avoid long-term consequences of MB.

## CONCLUSION

The experts who participated in the Delphi process considered that awake and habitual in preschoolers should be classified in three categories: NB, OB and MB. Children should be assessed primarily at rest. Time spent with an open mouth posture was considered as the most relevant sign to determine the breathing pattern. SLPs also reported the position of the lips and tongue as being essential to observe. The consensus highlights the fact that mouth posture while chewing and just after swallowing should complete the examination.

A clinical grid was developed based on this international consensus. This grid is intended to help the SLP make a nuanced decision, through a primary and secondary profile, on the awake habitual breathing pattern during the myofunctional assessment. While this grid seems a promising tool, further studies should explore its validity in comparison to an objective and reliable diagnosis tool.

## Appendix 1 List of the baseline items initially presented to the experts for reviewing

**Table:**

Level	Baseline items	References
A	**The breathing pattern classification should be split in three main and proper categories: Mouth breathing, nasal breathing or oronasal breathing**	Felicio et al., 2010 ; Felicio & Ferreira, 2008 ; Fujimoto et al., 2009 ; Grandi et al., 2012 ; Marchesan et al., 2012 ; Valera et al., 2003 ; Basheer et al., 2014 ; Sano et al., 2018 ; Yamaguchi et al., 2015 ; Ikenaga et al., 2013 ; Mattos, 2018 ; Milanesi et al., 2018 ; Andrade et al., 2012
A	**Watching a child breathing at rest in a spontaneous and stress-free context is relevant to determine the awake and habitual breathing pattern.**	Felicio et al., 2010 ; Felicio & Ferreira, 2008 ; Fujimoto et al., 2009 ; Ieto, 2011 ; Andrade et al., 2012
	Observing the child for 5 consecutive minutes at rest is enough to be relevant to determine the awake and habitual breathing pattern	Fujimoto et al., 2009 ; Nagaiwa et al., 2016; Ikenaga et al., 2013
B	Observing an open mouth posture at rest (in a spontaneous and stress-free context) is relevant to determine the awake and habitual breathing pattern	Basheer et al., 2014 ; Bueno et al., 2015 ; Cuccia et al., 2008 ; Harari et al., 2010 ; Junqueira et al., 2010 ; Lopes et al., 2014 ; Milanesi et al., 2018 ; Pacheco et al., 2015 ; Saitoh et al., 2018 ; Sano et al., 2018 ; Valera et al., 2003
B	Amplitude of mouth opening is relevant to determine the awake and habitual breathing pattern	Cattoni et al., 2007 ; Milanesi et al., 2018 ; Andrade et al., 2012
C	*Observing fully closed lips will influence your decision*	
C	*Observing the lips slightly open will influence your decision*	
C	*Observing half-open lips will influence your decision*	
C	*Observing wide open lips will influence your decision*	
C	*Observing that sometimes the mouth is open, sometimes the mouth is closed will influence your decision.*	
B	Time spent with an open mouth posture is relevant to determine the awake and habitual breathing pattern	Fujimoto et al., 2009
C	*Observing an open mouth posture (lips slightly, half or wide open) for the half of the observation time or more will influence your decision.*	
C	*Observing the mouth closed for the half of the observation time or more will influence your decision.*	
C	*Observing an open mouth posture for the entire observation time will influence your decision.*	
C	*Observing the mouth closed for the entire observation time will influence your decision.*	
B	Hearing noisy breathing at rest is relevant to determine the awake and habitual breathing pattern	Felcar et al., 2010 ; Marchesan, 2000 ;Valera et al., 2003
C	*Hearing nasal inspiration noises will influence your decision.*	
C	*Hearing oral inspiration noises will influence your decision.*	
B	Watching the child’s behaviors/habits at rest is relevant to determine the awake and habitual breathing pattern	Abreu et al., 2008 ; Lopes et al., 2014 ; Saitoh et al., 2018 ; Trawitzki et al., 2005 ; Valera et al., 2003
C	*Observing the child itching his/her nose will influence your decision.*	
C	*Observing the child licking and/or playing with his/her lips, or sticking out the tongue will influence your decision.*	
C	*Observing the child sucking his/her finger, lower lip or an object will influence your decision.*	
C	*Observing not any behaviors/habits will influence your decision.*	
C	*One occurrence of one of these behaviors during the entire observation is enough to be relevant to determine the awake and habitual breathing pattern*	
A	**Watching a child just after swallowing is relevant to determine the awake and habitual breathing pattern**	Bueno et al., 2015 ; Knösel et al., 2011 ; Saitoh et al., 2018 ; Valera et al., 2003
	Observing at least 2 sips is enough and relevant to determine the awake and habitual breathing pattern	
B	Observing the air intake just after swallowing is relevant to determine the awake and habitual breathing pattern.	
C	*Observing the child breathing through his/her mouth just after swallowing will influence your decision*	
C	*Observing the child breathing through his/her nose just after swallowing will influence your decision*	
C	*Observing a mouth opening just after swallowing will influence your decision.*	
C	*Observing the mouth closed just after swallowing will influence your decision.*	
A	**Watching the child while chewing is relevant to determine awake and habitual breathing pattern**	Felicio et al., 2010 ; Felicio & Ferreira, 2008 ; Saitoh et al., 2018 ; Silva et al., 2007 ; Valera et al., 2003 ; Ikenaga et al., 2013 ; Nagaiwa et al., 2016 ; Sano et al., 2018 ; Bueno et al., 2015
	Observing 2 bites is enough to be relevant to determine awake and habitual breathing pattern	
B	Time spent with an open mouth posture while chewing is relevant to determine the awake and habitual breathing pattern	
C	*Observing an open mouth posture for the half of the chewing occurrences or more will influence your decision.*	
C	*Observing the mouth closed for the half of the chewing occurrences or more will influence your decision.*	
C	*Observing an open mouth posture for all the chewing occurrences will influence your decision.*	
C	*Observing the mouth closed for all the chewing occurrences will influence your decision.*	
C	*Observing the child chewing with his/her mouth open will influence your decision.*	
C	*Observing the child chewing with his/her mouth closed will influence your decision.*	
A	**Encouraging or forcing the child to breathe through the nose (forced nasal breathing) is relevant to determine awake and habitual breathing pattern**	Bakke et al., 2007 ; Felicio et al., 2010 ; Felicio & Ferreira, 2008 ; Pacheco et al., 2015 ; Zaghi et al., 2020
	Placing tape on the child’s lips for 3 minutes is enough and relevant to determine awake and habitual breathing pattern	
	Verbally ask the child to take 5 consecutive breaths through the nose is enough and relevant to determine awake and habitual breathing pattern	
C	*Observing signs of tiredness while the child is forced to breathe through the nose will influence your decision.*	
C	*Observing signs of dyspnea while the child is forced to breathe through the nose will influence your decision.*	
C	*Observing signs of efforts to maintain labial closure (wrinkles on the chin, contraction of face muscles, …) when the child is forced to breathe through the nose will influence your decision.*	
C	*Observing none of the signs (tiredness, dyspnea, efforts) above will influence your decision.*	
C	*Observing that the child fails to breathe through the nose during the entire exercise will influence your decision.*	
B	Observing the air intake just after the forced breathing will influence your decision	

## Appendix 2 Expert’s comments on conditions

**Table:**

Baseline items	Items in the second questionnaire	Experts’ suggestions	Modified items according to experts’ suggestions
Observing the child for 5 consecutive minutes at rest is enough to be relevant to determine the awake and habitual breathing pattern	Observing the child for 3 consecutive minutes at rest in several different resting situations is relevant to determine the awake and habitual breathing pattern.	8/13 experts agreed with the proposal. The other mentioned essentiality and suggested modifications, such as increasing the number of observation opportunities rather than lengthen the observation time. They generally proposed between 2 and 4 different situations to observe: watching a film, stringing beads, drawing, playing, listening to a story. They also suggest observing the child at the beginning and end of the assessment.	Conditions of observation at rest: Observe the child for 3 consecutive minutes at rest **in 3 different resting situations (watching a movie, drawing, playing quietly, threading beads)** and at different moments of the assessment’s situation.
Observing at least 2 sips is enough and relevant to determine the awake and habitual breathing pattern	When observing the child’s air intake after swallowing, at least 3 sips of water are enough and relevant to determine the awake and habitual breathing pattern.	6/13 experts agreed with this proposal. The other mentioned essentiality and suggested modifications, such as increasing the number of sips. Most experts suggested up to 5 or 6 swallows, others suggested not to give a specific number but rather to offer a small glass of water.	Condition of observation of the child’s air intake after swallowing: **Observe the child drinking a small glass of water (at least 3 sips)** and watch the air intake after each swallow.
	When observing the child’s air intake after swallowing, at least 3 swallows of solid bolus are enough and relevant to determine the awake and habitual breathing pattern.	6/13 experts agreed with this proposal. The other mentioned essentiality and suggested modifications in line with the previous item: about 5 swallows or a whole food (like a biscuit).	Condition of observation of the child’s air intake after swallowing: **Observe the child eating 1 or 2 biscuits (at least 3 swallows)** and watch the air intake after each swallow.
Observing 2 bites is enough to be relevant to determine awake and habitual breathing pattern	When observing the child’s breathing during chewing, at least 3 bites are enough to be relevant to determine the awake and habitual breathing pattern.	6/13 experts agreed with this proposal. The other mentioned essentiality and suggested modifications in line with the previous item: about 5 bites or a whole food (like a biscuit).	Condition of observation of the child’s breathing during chewing: **Observe the child eating 1 or 2 biscuits (at least 3 bites).**

## Appendix 3 Details of each item and their respective CVR

**Table:**

	FIRST ROUND				SECOND ROUND (modified items)			
LEVEL	Items	N	CVR threshold	CVR	Items	N	CVR threshold	CVR
A	Watching a child breathing at rest in a spontaneous and stress-free context is relevant to determine the awake and habitual breathing pattern.	14	0.44	**0.57**	Validated in the first round			
	Observing the child for 5 consecutive minutes at rest is enough to be relevant to determine the awake and habitual breathing pattern	14	0.44	-0.86	Observing the child for 3 consecutive minutes at rest in several different resting situations is relevant to determine the awake and habitual breathing pattern	15	0.425	0.20
B	Observing an open mouth posture at rest (in a spontaneous and stress-free context) is relevant to determine the awake and habitual breathing pattern	14	0.44	0.00	Observing the mouth posture (lips and tongue) at rest is relevant to determine the awake and habitual breathing pattern	15	0.425	**0.60**
B	Amplitude of mouth opening is relevant to determine the awake and habitual breathing pattern	14	0.44	-0.29	Watching how open the lips are for more than half of the time at rest, is relevant to determine the awake and habitual breathing pattern	15	0.425	**0.47**
*C*								
*C*	*Observing fully closed lips will influence your decision*	7	0.622	0.43	*Observing fully closed lips for more than half of the time will influence your decision*	11	0.496	**0.82**
*C*								
	*Observing the lips slightly open will influence your decision*	7	0.622	-0.43	*Observing the lips slightly open for more than half of the time will influence your decision*	11	0.496	**0.64**
*C*								
	*Observing half-open lips will influence your decision*	7	0.622	-0.14	*Observing half-open lips for more than half of the time will influence your decision*	11	0.496	**1.00**
	*Observing wide open lips will influence your decision*	7	0.622	0.43	*Observing wide open lips for more than half of the time will influence your decision*	11	0.496	**0.64**
*C*								
	*Observing that sometimes the mouth is open, sometimes the mouth is closed will influence your decision.*	7	0.622	0.14	*Not observing a main pattern (sometimes the lips are open. sometimes the lips are closed) will influence your decision.*	11	0.496	**0.64**
B	Non-existent in first round				Observing the position occupied by the tongue for more than half of the time at rest is relevant to determine the awake and habitual breathing pattern	15	0.425	**0.73**
*C*					*Observing an upper tongue position for more than half of the time will influence your decision*	14	0.44	**0.86**
*C*					*Observing a low tongue position for more than half of the time will influence your decision*	14	0.44	**0.71**
*C*					*Observing a low and forward tongue position for more than half of the time will influence your decision*	14	0.44	**0.86**
*C*					*Not observing the tongue position (because of closed lips) for more than half of the time will influence your decision*	14	0.44	**0.57**
B	Time spent with an open mouth posture is relevant to determine the awake and habitual breathing pattern	14	0.44	**0.71**	Validated in the first round			
*C*	*Observing an open mouth posture (lips slightly, half or wide open) for the half of the observation time or more will influence your decision.*	13	0.456	**0.69**				
*C*	*Observing the mouth closed for the half of the observation time or more will influence your decision.*	13	0.456	**0.69**				
*C*	*Observing an open mouth posture for the entire observation time will influence your decision.*	13	0.456	**0.54**				
*C*	*Observing the mouth closed for the entire observation time will influence your decision.*	13	0.456	**0.85**				
B	Hearing noisy breathing at rest is relevant to determine the awake and habitual breathing pattern	14	0.44	-0.29	Knowing that the child does not present any medical condition, hearing noisy breathing at rest is relevant to determine the awake and habitual breathing pattern	15	0.425	-0.20
*C*	*Hearing nasal inspiration noises will influence your decision.*	9	0.548	-0.11	*Knowing that the child does not present any medical condition, hearing nasal inspiration noises on repeated breathing cycles will influence your decision.*	11	0.496	0.27
*C*	*Hearing oral inspiration noises will influence your decision.*	9	0,548	-0.33	*Knowing that the child does not present any medical condition, hearing oral inspiration noises on repeated breathing cycles will influence your decision.*	11	0.496	0.27
B	Watching the child’s behaviors/habits at rest is relevant to determine the awake and habitual breathing pattern	14	0.44	**0.71**	Validated in the first round			
	One occurrence of one of these behaviors during the entire observation is enough to be relevant to determine the awake and habitual breathing pattern *(ex. Observing the child sticking out his/her tongue just once is enough to be relevant to determine awake and habitual breathing pattern)*	13	0.456	-0.85	In addition to other the signs, observing more than one occurrence of some habits/behaviors (finger sucking, nose itching, lips playing) during the entire observation is relevant to determine the awake and habitual breathing pattern	15	0.425	0.33
*C*	*Observing the child itching his/her nose will influence your decision.*	13	0.456	-0.69	*In addition to the other signs, observing the child itching his/her nose on more than one occasion will influence your decision.*	11	0.496	-0.09
*C*	*Observing the child licking and/or playing with his/her lips, or sticking out the tongue will influence your decision.*	13	0,456	-0.23	*In addition to the other signs, observing the child licking and/or playing with his/her lips, or sticking out the tongue on more than one occasion will influence your decision.*	11	0.496	**0.82**
*C*	*Observing the child sucking his/her finger, lower lip or an object will influence your decision.*	13	0.456	-0.38	*In addition to the other signs, observing the child sucking his/her thumb, sucking his/her lower lip or sucking an object on more than one occasion will influence your decision.*	11	0.496	**0.82**
*C*	*Observing not any behaviors/habits will influence your decision.*	13	0.456	-0.54	*In addition to the other signs, not observing any behaviors/habits will influence your decision.*	11	0.496	**0.82**
A	Watching a child just after swallowing is relevant to determine the awake and habitual breathing pattern	14	0.44	**0.57**	Validated in the first round			
	Observing at least 2 sips is enough and relevant to determine the awake and habitual breathing pattern	11	0.496	-0.09	When observing the child’s air intake after swallowing, at least 3 sips of water are enough and relevant to determine the awake and habitual breathing pattern	15	0.425	-0.07
	Non-existent in first round				When observing the child’s air intake after swallowing, at least 3 swallows of solid bolus are enough and relevant to determine the awake and habitual breathing pattern	15	0.425	-0.07
B	Observing the air intake just after swallowing is relevant to determine the awake and habitual breathing pattern.	11	0.496	**0.64**
*C*	*Observing the child breathing through his/her mouth just after swallowing will influence your decision*	10	0.52	**0.60**	Validated in the first round			
*C*	*Observing the child breathing through his/her nose just after swallowing will influence your decision*	10	0.52	**0.80**
*C*	*Observing a mouth opening just after swallowing will influence your decision.*	11	0.496	0.09	*When watching the child’s air intake after swallowing, observing a mouth opening in most cases will influence your decision.*	15	0.425	**0.87**
*C*	*Observing the mouth closed just after swallowing will influence your decision.*	11	0.496	**0.64**				

A	Watching the child while chewing is relevant to determine the awake and habitual breathing pattern	14	0.44	**0.57**	Validated in the first round			
	Observing 2 bites is enough to be relevant to determine the awake and habitual breathing pattern	12	0.475	-0.50	When observing the child’s breathing during chewing. at least 3 bites are enough to be relevant to determine the awake and habitual breathing pattern	15	0.425	-0.33
*C*	*Observing the child chewing with his/her mouth open will influence your decision.*	12	0.475	**0.83**	Validated in the first round			
*C*	*Observing the child chewing with his/her mouth closed will influence your decision.*	12	0.475	**0.50**				
B	Time spent with an open mouth posture while chewing is relevant to determine the awake and habitual breathing pattern	12	0.475	**0.83**				
*C*	*Observing an open mouth posture for the half of the chewing occurrences or more will influence your decision.*	11	0.496	**0.82**				
*C*	*Observing the mouth closed for the half of the chewing occurrences or more will influence your decision.*	11	0.496	**0.64**				
*C*	*Observing an open mouth posture for all the chewing occurrences will influence your decision.*	11	0.496	**1.00**				
*C*	*Observing the mouth closed for all the chewing occurrences will influence your decision.*	11	0.496	**0.64**				
A	Encouraging or forcing the child to breathe through the nose (forced nasal breathing) is relevant to determine the awake and habitual breathing pattern	14	0.44	0.14	In addition to the other contexts of observation, encouraging or forcing the child to breathe through the nose (forced nasal breathing) is relevant to determine the awake and habitual breathing pattern	15	0.425	0.33
	Placing tape on the child’s lips for 3 minutes is enough and relevant to determine the awake and habitual breathing pattern	10	0.52	-0.60	In addition to the other signs or assessment situations, placing tape on the child’s lips for 3 minutes is enough and relevant to determine the awake and habitual breathing pattern	13	0.496	-0.69
	Verbally ask the child to take 5 consecutive breaths through the nose is enough and relevant to determine the awake and habitual breathing pattern	10	0.52	-0.20	In addition to the other signs or assessment situations, verbally asking the child to take 7 consecutive breaths through the nose is enough and relevant to determine the awake and habitual breathing pattern	13	0.496	-0.23
*C*	*Observing signs of tiredness while the child is forced to breathe through the nose will influence your decision.*	10	0.52	**0.60**	Validated in the first round			
*C*	*Observing signs of dyspnea while the child is forced to breathe through the nose will influence your decision.*	10	0.52	**0.60**				
*C*	*Observing signs of efforts to maintain labial closure (wrinkles on the chin. contraction of face muscles, …) when the child is forced to breathe through the nose will influence your decision.*	10	0.52	**0.80**				
*C*	*Observing none of the signs (tiredness, dyspnea, efforts) above will influence your decision.*	10	0.52	0.00	*In addition to the other signs or assessment situations, not observing any sign of tiredness, dyspnea or effort when the child is forced to breathe through the nose will influence your decision.*	13	0.496	0.38
*C*	*Observing that the child fails to breathe through the nose during the entire exercise will influence your decision.*	10	0.52	**0.80**	Validated in the first round			
B	Observing the air intake just after the forced breathing will influence your decision	10	0.52	**0.60**				

S-CVI average round 1= 0.63 S-CVI average round 2= 0.6 S-CVI total average = 0.77;

Bold items are CVR > threshold
